# Educational Levels and Risk of Suicide in Japan: The Japan Public Health Center Study (JPHC) Cohort I

**DOI:** 10.2188/jea.JE20140253

**Published:** 2016-06-05

**Authors:** Takashi Kimura, Hiroyasu Iso, Kaori Honjo, Satoyo Ikehara, Norie Sawada, Motoki Iwasaki, Shoichiro Tsugane

**Affiliations:** 1 Public Health, Department of Social Medicine, Osaka University Graduate School of Medicine, Suita, Osaka, Japan; 1 大阪大学大学院医学系研究科社会医学講座公衆衛生学; 2 Global Collaboration Center, Osaka University, Suita, Osaka, Japan; 2 大阪大学グローバルコラボレーションセンター; 3 Department of Hygiene and Public Health, Osaka Medical College, Takatsuki, Osaka, Japan; 3 大阪医科大学衛生学・公衆衛生学; 4 Epidemiology and Prevention Group, Research Center for Cancer Prevention and Screening, National Cancer Center, Tokyo, Japan; 4 国立がん研究センターがん予防・検診研究センター

**Keywords:** suicide, education, socioeconomic status, prospective study, 自殺, 教育歴, 社会経済的地位, 前向き研究

## Abstract

**Background:**

Suicide rates have been related to educational level and other socioeconomic statuses. However, no prospective study has examined the association between educational level and the risk of suicide in Japan.

**Methods:**

We examined the association of education level and suicide risk in a population-based cohort of Japanese men and women aged 40–59 years in the Japan Public Health Center-based Prospective Study Cohort I. In the baseline survey initiated in 1990, a total of 46 156 subjects (21 829 men and 24 327 women) completed a self-administered questionnaire, which included a query of educational level, and were followed up until the end of December 2011. Educational levels were categorized into four groups (junior high school, high school, junior or career college, and university or higher education). During a median follow-up of 21.6 years, the hazard ratios (HRs) and 95% confidence intervals (CIs) of suicide according to educational level were estimated using the Cox proportional hazards regression model adjusted for age; study area; previous history of stroke, ischemic heart disease, or cancer; self-reported stress; alcohol consumption; smoking; living with spouse; and employment status. A total of 299 deaths attributed to suicide occurred.

**Results:**

The HR for university graduates or those with higher education versus junior high school graduates was 0.47 (95% CI, 0.24–0.94) in men, and that for high school graduates versus junior high school graduates was 0.44 (95% CI, 0.24–0.79) in women.

**Conclusions:**

High educational levels were associated with a reduced risk of suicide for both Japanese men and women.

## Abstract (ja)

**【背景】:**

自殺率は教育歴やその他の社会経済的地位と関係するとされている。しかしながら、日本においては、教育歴と自殺リスクとの関連をみた前向き研究はこれまでされてこなかった。

**【方法】:**

多目的コホート研究のコホートIに参加した40-59歳の男性と女性について関連性を検討した。1990年に開始されたベースライン調査において教育歴を含むアンケートに回答し、かつ2011年12月まで追跡可能な者46156人（男性21829人、女性24327人）を対象とした。教育歴は最終教育歴を4つのレベル（中学、高校、短大及び専門学校、大学及びそれ以上）に区分した。 21.6年間の追跡期間の、教育歴に応じた自殺のハザード比について、年齢、調査地域、脳卒中・虚血性心疾患・がんの既往歴、自己申告のストレス、アルコール摂取量、喫煙歴、配偶者との同居、雇用状況を調整し、コックス比例ハザード回帰モデルを用いて算出した。自殺に起因する299人の死亡の合計が発生した。

**【結果】:**

男性では、大学及びそれ以上の教育歴の者は、中卒の者に対してのHRは0.47（0.24-0.94）であった。女性では高卒の者は、中卒の者に対してのHRは0.44（0.24-0.79）であった。

**【結論】:**

日本人の男女いずれにおいても、高教育歴は自殺のリスク低下と関連していた。

## INTRODUCTION

Suicide is a serious global public health issue. Every year, more than 800 000 people die from suicide.^1^ In Japan, from 1996 to 2012, the death rate from suicide was approximately 20 per 100 000 individuals, with the highest rate in 1998 of over 50 per 100 000 among men aged 45 to 64 years.^2^

Previous studies have indicated that the risk of suicide varies with socioeconomic status,^3^^–^^7^ especially with respect to income,^8^ occupation,^9^^,^^10^ living arrangement,^11^ and educational level.^12^^–^^15^ These associations suggest that suicide is induced by socioeconomic factors as well as biological, psychological, and lifestyle factors and the presence of illness. We considered educational level to serve as a surrogate marker for socioeconomic status. In general, college graduates or those with advanced degrees gain greater public respect compared to those with less education, regardless of their occupational positions.^16^ Still, educational levels are correlated strongly with occupation and income and remain stable over an individual’s lifetime.^17^ Effects from an individual’s economic change or the shifting of employment status in later life may be small due to the relatively stable employment system in Japan, but such a situation is not necessarily true in other countries.

However, research on the association between an individual’s educational level and suicide risk has been limited and inconclusive. According to a previous cohort study in the United States, compared with higher levels of education, high school graduation and lower levels of education are associated with a higher risk of suicide in men but not in women, after adjustment for race, ethnicity, geographic variables, marital status, and employment status.^12^ A European comparative cohort study indicated that the lower educational categories (International Standard Classification of Education [ISCED] 1 and 2) are generally associated with a higher risk of suicide than the higher categories (ISCED 3+) in men, whereas the higher categories (ISCED 3+) are weakly but significantly associated with a higher risk of suicide in women.^13^

An Italian nationwide registry study showed a significant difference in the educational attainments between suicide victims and persons with natural causes of death. For both men and women, persons who died from suicide were more likely to have higher educational attainment than those who died from natural causes.^14^ However, this finding should be interpreted with caution, because deaths from natural causes were used as a reference. On the other hand, a Japanese autopsy study, using 145 gender-, age-, and municipality-matched living controls, showed that 28.6% of suicide completers and 16.6% of living controls had low educational attainment (≤11 years).^15^ These inconsistent results may be in part due to different educational and occupational systems among countries.

In response to the discrepancy of previous findings, we examined the association between educational levels and the risk of suicide in a large prospective cohort study of approximately 45 000 Japanese adults.

## METHODS

### Study cohort

The Japan Public Health Center-based Prospective Study (JPHC study) is a population-based cohort study conducted since 1990 among 61 595 individuals (29 980 men and 31 615 women aged 40 to 59 years) who registered their addresses in 15 administrative districts supervised by five public health center areas in Cohort I. The study design has been described in detail elsewhere.^16^

A total of 23 584 men (79%) and 26 661 women (84%) responded to the baseline questionnaire. We excluded 28 people because of ineligibility (7 non-Japanese and 21 Japanese who moved away before the start of the study). Additionally, we excluded 4061 people (1742 men and 2319 women) who did not report valid information on educational level from the analysis. Ultimately, 46 156 subjects (21 829 men and 24 327 women) were used for the present analysis. Information on the study purpose and methods for the baseline and follow-up is disclosed on the JPHC website (http://epi.ncc.go.jp/jphc/). The study protocol was approved by the Institutional Review Board of the National Cancer Center and the Osaka University, Japan.

### Assessment of educational levels and covariates

The study participants completed a self-administered questionnaire, which included a query of educational levels. The Japanese educational system consists of 6 years of elementary school and 3 years of junior high school, which are compulsory education. As for elective education, there are 3 years of high school, 2 years of junior college, 2 or 3 years of career college, and 4 years of university. In our study, educational levels were categorized as graduation from junior high school, high school, junior college, career college, and university or higher education. We also inquired about personal and familial medical histories, smoking habit, habitual intake of foods and beverages (including alcohol), physical activity, perceived stress levels, living arrangement, occupation, and other lifestyle factors.

### Follow-up and identification of suicide

The study participants were followed from the start of the study in 1990 until December 31, 2011. The residential registry in each area was reviewed annually to obtain information on changes in residential status, including survival. The status of subjects who had moved out of the study area was assessed through the municipal office of the area to which they had moved. Mortality data for persons under the residential registry were forwarded to the Ministry of Health, Labour and Welfare and coded for inclusion in the National Vital Statistics.

Information on the deaths of the subjects who remained in the original area was obtained from local public health centers (PHCs); information on the deaths of the subjects who died after moving from their original PHC area was obtained from death certificates maintained by the Ministry of Health, Labour and Welfare, Japan. The cause of death was also obtained from the Ministry of Health, Labour and Welfare with the permission of the Ministry of Internal Affairs and Communications. Suicidal death was defined according to the International Classification of Diseases, 10th Revision (ICD-10; codes X60 to X84).

### Statistical analysis

The number of person-years in the follow-up period was calculated from the date of response to the baseline questionnaire to the date of death or December 31, 2011, whichever came first. The hazard ratios (HRs) and 95% confidence intervals (CIs) were calculated according to the categories of graduates from high school, junior or career college, and university or higher education, with junior high school graduates as a reference, using Cox proportional-hazard models. The statistical power under the condition of α = 0.05 (two-tailed) and an HR of 0.5 was 80.1%.

We calculated HRs of suicide adjusting for age and study areas. Then, we adjusted further for previous history of stroke, ischemic heart disease, or cancer (yes/no); self-reported stress (mild, moderate, or high); alcohol consumption (non-, occasional, or current drinker); smoking habit (never, ex-, or current smoker); living with spouse (yes/no); and full-time worker (yes/no). We also calculated the HRs of suicide after the exclusion of persons with a previous history of stroke, ischemic heart disease, or cancer. To examine whether preclinical disorders and economic recession during the follow-up affected the association between educational levels and the risk of suicide, we calculated the HRs of suicide after the exclusion of suicidal deaths that occurred within 1 to 10 years of the baseline survey.

We tested the trends across educational levels using the ordinal numbers 0 to 3 assigned to the categories of educational levels. All statistical analyses were performed with SAS software version 9.4 (SAS Institute, Inc., Cary, NC, USA). All reported *P*-values were two-sided, and the significance level was set at *P* < 0.05.

## RESULTS

The sex-specific distributions of baseline characteristics according to the four categories of educational level are presented in Table 1. The participants were composed of 47.4% junior high school graduates, 38.7% high school graduates, 4.9% junior college graduates, and 9.0% university graduates or those with higher education in men; for women, the proportions were 51.9%, 36.6%, 9.5%, and 2.1%, respectively. Both men and women with higher educational levels were younger; more likely to have high perceived mental stress, live with a spouse, and be full-time workers; and less likely to be current smokers. Current drinkers were less common among junior high school graduates than among higher education graduates. Men with higher educational levels were less likely to have a previous history of stroke, ischemic heart disease, or cancer.

**Table 1.** Distributions of baseline characteristics according to educational levels

	Educational levels				

	Junior high school	High school	Junior or career college	University or higher education	*P* for trend
Men					
Number of subjects	10 350	8437	1074	1968	
Age, years	50.5	48.1	47.5	46.7	<0.001
Previous history of stroke, ischemic heart disease, or cancer, %	3.6	3.0	2.8	2.3	<0.001
Current drinker, %	64.3	69.3	65.1	65.9	0.006
Current smoker, %	54.9	53.1	51.0	47.0	<0.001
High perceived mental stress, %	20.0	29.3	33.9	43.8	<0.001
Living with spouse, %	76.0	86.4	87.9	90.6	<0.001
Full-time worker, %	96.0	97.4	97.8	97.8	<0.001

Women					
Number of subjects	12 624	8894	2305	504	
Age, years	50.7	48.0	46.3	45.7	<0.001
Previous history of stroke, ischemic heart disease, or cancer, %	4.1	3.3	3.9	3.1	0.08
Current drinker, %	9.0	11.0	11.0	10.2	<0.001
Current smoker, %	7.6	6.8	6.5	6.5	0.02
High perceived mental stress, %	18.5	22.8	29.3	40.9	<0.001
Living with spouse, %	69.6	81.6	81.6	83.2	<0.001
Full-time worker, %	74.0	76.7	76.5	85.9	<0.001

A total of 299 (218 men and 81 women) who died by suicide were documented during the median follow-up of 21.6 years. Table 2 shows sex-specific HRs of suicide according to educational levels. Age- and area-adjusted risk of suicide was lower in men with university graduation or higher education than in those with junior high school education. The multivariable HRs of suicide for university graduates or those with higher education compared with those of junior high school graduates after further adjustment for other potential confounding variables were 0.47 (95% CI, 0.24–0.94) for all men, 0.43 (95% CI, 0.17–1.08) for men aged 40 to 49 years, and 0.64 (95% CI, 0.23–1.78) for men aged 50 to 59 years. The corresponding HRs after the exclusion of persons with a previous history of stroke, ischemic heart disease, or cancer at the baseline survey were 0.48 (95% CI, 0.24–0.97), 0.44 (95% CI, 0.17–1.10), and 0.67 (95% CI, 0.24–1.89).

**Table 2.** Sex-specific hazard ratios of suicide according to educational levels

	Men				Women			
	
	Junior high school	High school	Junior or career college	University or higher education	Junior high school	High school	Junior or career college	University or higher education
All ages of 40–59 years								
Person-years	202 414	168 847	21 517	39 276	259 699	182 585	47 301	10 257
Number of suicides	119	79	11	9	60	15	5	1
Hazard ratio (95% CI)^a^	1.00	0.85 (0.64–1.15)	0.95 (0.51–1.76)	0.43 (0.22–0.85)	1.00	0.44 (0.24–0.79)	0.58 (0.23–1.46)	0.57 (0.08–4.15)
Hazard ratio (95% CI)^b^	1.00	0.91 (0.67–1.22)	1.01 (0.54–1.90)	0.47 (0.24–0.94)	1.00	0.44 (0.24–0.79)	0.56 (0.22–1.43)	0.55 (0.08–4.03)

Ages 40–49 years								
Person-years	83 716	96 233	13 349	25 767	100 700	101 890	31 399	7539
Number of suicides	54	50	8	5	18	4	3	—
Hazard ratio (95% CI)^a^	1.00	0.89 (0.60–1.32)	1.16 (0.55–2.46)	0.39 (0.15–0.97)	1.00	0.21 (0.07–0.63)	0.51 (0.15–1.79)	—
Hazard ratio (95% CI)^b^	1.00	0.92 (0.62–1.38)	1.26 (0.59–2.69)	0.43 (0.17–1.08)	1.00	0.21 (0.07–0.65)	0.51 (0.14–1.81)	—

Ages 50–59 years								
Person-years	118 698	72 615	8168	13 509	158 999	80 695	15 902	2718
Number of suicides	65	29	3	4	42	11	2	1
Hazard ratio (95% CI)^a^	1.00	0.81 (0.52–1.27)	0.69 (0.22–2.21)	0.58 (0.21–1.61)	1.00	0.61 (0.31–1.21)	0.51 (0.12–2.14)	1.40 (0.19–10.2)
Hazard ratio (95% CI)^b^	1.00	0.87 (0.55–1.38)	0.70 (0.22–2.26)	0.64 (0.23–1.78)	1.00	0.60 (0.30–1.19)	0.49 (0.12–2.03)	1.30 (0.18–9.60)

CI, confidence interval.

^a^Adjusted for age and study areas.

^b^Adjusted further for previous history of stroke, ischemic heart disease, or cancer (yes/no), perceived mental stress (mild, moderate, or severe), alcohol consumption (nondrinker, occasional drinker, or current drinker), smoking status (never, ex-, or current smoker), living with spouse (yes/no), and full-time worker (yes/no).

Age- and area-adjusted risk of suicide was lower in women who graduated high school than in those who only graduated junior high school. The multivariable HRs of suicide for high school graduates compared with those of junior high school graduates after further adjustment for other potential confounding variables were 0.44 (95% CI, 0.24–0.79) for all women, 0.21 (95% CI, 0.07–0.65) for women aged 40 to 49 years, and 0.60 (95% CI, 0.30–1.19) for women aged 50 to 59 years. The corresponding HRs after the exclusion of persons with a previous history of stroke, ischemic heart disease, or cancer at the baseline survey were 0.41 (95% CI, 0.22–0.76), 0.21 (95% CI, 0.07–0.65), and 0.56 (95% CI, 0.26–1.15).

Figure illustrates the sex-specific HRs of suicide for those with higher education after the exclusion of early suicidal deaths that occurred within 1 to 10 years of the baseline survey. Compared with junior high school graduates, male university graduates or those with higher education and female high school graduates had a persistently lower risk of suicide.

**Figure.**
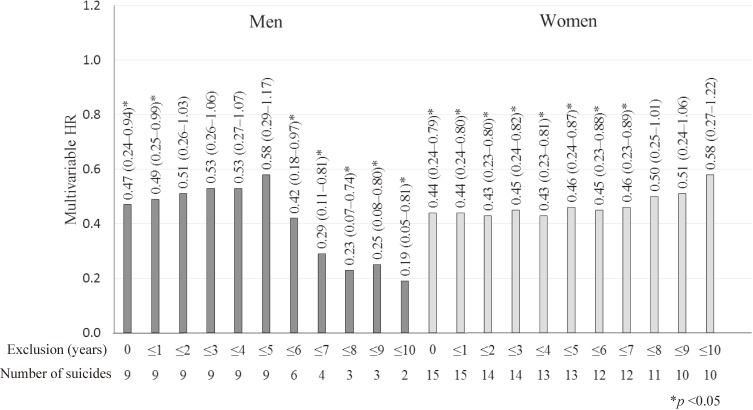
The sex-specific HRs and 95% CIs of suicide for those with higher education compared to those with junior high school graduates after the exclusion of early suicidal deaths that occurred within 1 to 10 years of the baseline survey. CI, confidence interval; HR, hazard ratio.

## DISCUSSION

In this large prospective study, we found that higher educational levels were associated with a lower risk of suicide for both men and women. The risk of suicide was approximately 50% lower among male university graduates or those with higher education than among male junior high school graduates. The risk of suicide was approximately 60% lower among female high school graduates than among female junior high school graduates. The risk difference by educational levels was more pronounced for men and women aged 40 to 49 years than for those aged 50 to 59 years. To our knowledge, this is the first population-based prospective study in Japan to examine the association between educational levels and the risk of suicide after controlling for individual lifestyles and socioeconomic factors.

Depression is a well-known risk factor for suicide.^17^^–^^19^ Unfortunately, we did not have any information on depression; however, we did collect information on mental stress. Since persons with higher educational levels were likely to have higher perceived mental stress in the present study, the association between higher educational levels and a lower risk of suicide was unlikely to be explained by mental stress. Moreover, we analyzed the risk of suicide after the exclusion of early suicidal deaths that occurred within 1 to 10 years of the baseline survey; however, the association between higher educational levels and a lower risk of suicide did not change substantially when these suicidal deaths were excluded.

During the follow-up, major recessions in Japan occurred between 1991 and 1993 and between 1997 and 1999.^20^ The rate of suicide increased temporally among middle-aged men between 1998 and 2011.^2^^,^^21^ In the present study, however, the HRs of suicide after the exclusion of suicidal deaths that occurred within 1 to 10 years of the baseline survey did not change substantially. This finding suggests that the economic recessions were unlikely to have influenced the association between educational levels and the risk of suicide in our study.

Although the mechanisms behind the association between educational levels and the risk of suicide are not clear, a possible explanation can be supposed. Low serum cholesterol levels have been shown to be associated with the risk of suicide, likely through lower serotonin levels in blood and brain circulation.^22^^–^^24^ This explanation is supported by our finding that men with higher educational levels had higher age-adjusted mean values of total serum cholesterol than male junior high school graduates; in our subsamples of men (*n* = 6988), mean cholesterol levels were 194 mg/dL for junior high school graduates, 195 mg/dL for high school graduates, 195 mg/dL for junior or career college graduates, and 200 mg/dL for university graduates or those with higher education (*P* for difference <0.001); however, this explanation would not be applicable to women because the respective mean values of cholesterol among women (*n* = 11 663) in our study were 201 mg/dL, 203 mg/dL, 203 mg/dL, and 199 mg/dL (*P* for difference <0.001).

Our findings are consistent with the results from a Japanese autopsy study,^15^ a European comparative study (concerning men),^13^ and an American cohort study,^12^ but not with the results from an Italian nationwide register study (concerning both men and women)^14^ and the European comparative study (concerning women),^13^ which showed that higher educational levels are associated with a higher risk of suicide.

Until recently, Japanese women have generally dropped their careers to raise children or to concentrate on domestic duties.^25^ Therefore, they are unlikely to be influenced by their own employment status or income, and are more likely to be influenced by educational levels as the primary exposure of socioeconomic status. The discrepancy between the results of our study and those of the Italian study^14^ may be explained in part by the limited comparability with the Italian nationwide register study, as described in the introduction. The discrepancy may also be explained by the different employment systems between Japan and Italy. The employment system in Japan in the 1990s was primarily based on lifetime employment and seniority, especially for workers with higher educational levels. Italian workers with higher educational levels, however, may have had stronger psychological reactions under higher mental stress than those with lower educational levels.^14^ Different impacts of educational levels on health due to race or ethnicity, although not identified, could also have led to the discrepancy.^26^^,^^27^

The major strengths of the present study are its population-based prospective design, large sample size, and adjustment for potential confounding variables. However, there are several limitations. First, the number of suicides was small, but the expected statistical power was acceptable for men (although not for women). Second, our subjects were overall less educated compared with national samples because our surveyed communities were primarily located in rural areas. In our study population, the educational levels were composed of 50% junior high school graduates, 38% high school graduates, 7% junior college graduates, and 5% university graduates or those with higher education, while the corresponding proportions for national samples are 34%, 49%, 6%, and 11%.^28^ Therefore, it is uncertain whether our findings are generalizable to urban populations, and further studies in urban populations or nationally representative samples are needed. Third, the educational system in Japan changed in 1947. After World War II, the Fundamental Law of Education and the School Education Law forced the educational system to change from dual-track to single-track. Therefore, we analyzed the data stratified by age 40 to 49 and 50 to 59. For men and women, the inverse association between educational levels and the risk of suicide tended to be more evident in ages 40 to 49 than in ages 50 to 59. This finding may be explained by the large heterogeneity of educational levels in each educational category in the previous dual-track system, which may have diluted the association between educational levels and the risk of suicide. Fourth, we did not have data on mental illness. Someone who had developed a mental illness in early adolescence (eg, schizophrenia) would have been unlikely to obtain higher education, but it would also have been unlikely for them to participate in our study at the ages of 40 to 69 years. Finally, we did not have data on income levels, home ownership, and other economic factors. A European comparative study indicated that the lack of home ownership is more strongly associated with the risk of suicide than low educational levels.^13^ Among our study participants, however, the number of those lacking home ownership may have been very small.

In conclusion, high educational levels among Japanese men and women are associated with a reduced risk of suicide, suggesting that higher educational levels may have a protective role against suicide risk in Japanese society.

## ONLINE ONLY MATERIAL

Abstract in Japanese.
