# Supplementary material for: Educational Levels and Risk of Suicide in Japan: The Japan Public Health Center Study (JPHC) Cohort I
Source: J Epidemiol. 2016 Jun 5;26(6):315–21. doi: 10.2188/jea.JE20140253 (PMC4884900; doi:10.2188/jea.JE20140253)
Supplement: Abstract in Japanese. [file je-26-315-s001.pdf]

教育歴と自殺について：多目的コホート研究

木村尚史[1]、磯博康[1]、本庄かおり[2]、池原賢代[1,3]、澤田典絵[4]、岩崎基[4]、津金昌一郎[4]、
the JPHC Study Group

[1] 大阪大学大学院医学系研究科社会医学講座公衆衛生学
[2] 大阪大学グローバルコラボレーションセンター
[3] 大阪医科大学衛生学・公衆衛生学
[4] 国立がん研究センターがん予防・検診研究センター

【背景】自殺率は教育歴やその他の社会経済的地位と関係するとされている。しかしながら、日本においては、教育歴と自殺リスクとの関連をみた前向き研究はこれまでされてこなかった。

【方法】多目的コホート研究のコホートⅠに参加した 40-59 歳の男性と女性について関連性を検討した。1990 年に開始されたベースライン調査において教育歴を含むアンケートに回答し、かつ 2011 年 12 月まで追跡可能な者 46156 人（男性 21829 人、女性 24327 人）を対象とした。教育歴は最終教育歴を 4 つのレベル（中学、高校、短大及び専門学校、大学及びそれ以上）に区分した。 21.6 年間の追跡期間の、教育歴に応じた自殺のハザード比について、年齢、調査地域、脳卒中・虚血性心疾患・がんの既往歴、自己申告のストレス、アルコール摂取量、喫煙歴、配偶者との同居、雇用状況を調整し、コックス比例ハザード回帰モデルを用いて算出した。自殺に起因する 299 人の死亡の合計が発生した。

【結果】男性では、大学及びそれ以上の教育歴の者は、中卒の者に対しての HR は 0.47(0.24-0.94）であった。女性では高卒の者は、中卒の者に対しての HR は 0.44（0.24-0.79）であった。

【結論】日本人の男女いずれにおいても、高教育歴は自殺のリスク低下と関連していた。

キーワード：自殺、教育歴、社会経済的地位、前向き研究
